# Advances in mechanical force-induced adaptive responses and pathological transition of renal tubular epithelial cells

**DOI:** 10.3389/fcell.2026.1981323

**Published:** 2026-09-30

**Authors:** Shilin Li, Yuefeng Cai, Yi Zeng, Zhixia Song, Xisheng Wang

**Affiliations:** 1 The Longhua Affiliated Hospital (People's Hospital of Longhua), School of Medicine, Southern University of Science and Technology, Shenzhen Longhua School of Clinical Medicine, Guangdong Medical University, Shenzhen, China; 2 Peking University Shenzhen Hospital, Shenzhen Xin Hua Hospital, Shenzhen, China

**Keywords:** adaptive response, mechanical stimulation, obstructive nephropathy, pathological transition, renal tubular epithelial cells

## Abstract

Renal tubular epithelial cells (RTECs) reside in a mechanical microenvironment shaped by fluid shear stress, hydrostatic pressure, and cyclic stretch. Under physiological conditions, moderate mechanical stimulation helps maintain normal renal function by regulating cell volume, ion transport, and transcriptional programs. Tubular injury occurs when the intensity or duration of stimulation exceeds physiological tolerance. The balance between protective repair and pathological injury is determined jointly by stimulus intensity, exposure duration, and cellular adaptive capacity. Existing reviews of mechanical stress-induced kidney injury have largely emphasized pathological endpoints. The distinctive contribution of this review is to place the adaptive-to-pathological transition (the threshold at which protective mechanisms fail) and the segmental heterogeneity of tubular mechanoresponses at the center of its framework. We synthesize the molecular mechanisms by which RTECs sense and transduce mechanical signals, with particular emphasis on early adaptive responses including cytoskeletal reorganization, protein quality control, and remodeling of ion homeostasis, and we describe how persistent mechanical stimulation drives the transition from physiological adaptation to pathological injury. We further discuss quantitative and contextual thresholds for this transition, clinically relevant mechanical scenarios beyond classical obstruction, and the phase dependence of mechanosensitive therapeutic targets such as YAP/TAZ. Current research models and technologies are reviewed together with unresolved questions and future directions. By framing mechanically driven kidney disease as a continuum between adaptation and injury, this synthesis provides a theoretical basis for mechanistic studies and for timing-aware, segment-specific clinical translation in mechanical force-related kidney diseases.

## 1 Introduction

Renal tubular epithelial cells (RTECs) are among the kidney cell types most sensitive to mechanical stimulation and most extensively exposed to mechanical load (Ferrell et al., 2019; Choudhury et al., 2022). Under normal physiological conditions, these cells continuously encounter several mechanical signals. They include fluid shear stress generated by luminal flow, transmural hydrostatic pressure across blood vessels and renal tubules, and cyclic stretch caused by respiration and the heartbeat (Ferrell et al., 2019; Choudhury et al., 2022). Together, these forces create the mechanical microenvironment in which tubular cells reside. They help maintain normal tubular architecture and regulate cell polarity, ion transport, metabolism, and gene expression. Mechanical forces are therefore important physiological determinants of renal homeostasis (Nauryzgaliyeva et al., 2023; Tabibzadeh et al., 2023; Lee et al., 2021).

Mechanical forces produce bidirectional effects on tubular cells (Adapala et al., 2021; Liu et al., 2022). At physiological levels, they sustain polarity, cytoskeletal integrity, and transporter function, supporting reabsorption and secretion. Supraphysiological or prolonged stimulation, however, switches these responses toward stress, inflammation, and fibrotic injury (Liu et al., 2022; Zhao B. et al., 2022).

Clinically, ureteral obstruction, congenital urinary tract obstruction, polycystic kidney disease, and high-irrigation conditions during ureteroscopy or percutaneous nephrolithotomy can cause a sustained rise in intratubular pressure. RTECs are then exposed to prolonged abnormal stretch and high hydrostatic pressure, eventually resulting in tubular injury and interstitial fibrosis (Gilmer et al., 2018; Huang et al., 2023; Hadpech and Thongboonkerd, 2024; Cao et al., 2023). These obstructive conditions, however, represent only part of the clinical spectrum, because intratubular pressure and flow are dynamically dictated by the balance between glomerular filtrate production and downstream tubular reabsorption. Glomerular hyperfiltration in early diabetic nephropathy or after nephrectomy increases ultrafiltrate volume and flow, raising tubular wall tensile stress and fluid shear stress even in the absence of anatomical obstruction (Chagnac et al., 2019; Vallon and Thomson, 2020). Osmotic diuresis, in which non-reabsorbed solutes retain water within the lumen, likewise expands luminal volume, increases flow rate, and elevates secondary intratubular hydraulic pressure (Vallon and Thomson, 2020). Conversely, extrinsic mechanical compression can raise interstitial and intratubular pressure: severe interstitial edema during acute pyelonephritis or intense inflammation compresses tubular walls, and extreme visceral adiposity with perirenal fat accumulation and elevated intra-abdominal pressure imposes additional loads on the renal parenchyma (Mende and Einhorn, 2022; Abe et al., 2023). Integrating these hemodynamic and extrinsic scenarios provides a more balanced pathophysiological framework for mechanically mediated tubular injury. Mechanical forces are therefore not only initiating factors in kidney injury but also act throughout disease onset, progression, and repair (Zhao X. et al., 2022; Grabias and Konstantopoulos, 2013; Lee et al., 2015; Bai et al., 2022).

RTECs convert external physical stimuli into intracellular biochemical signals through several mechanosensors, including mechanosensitive ion channels, primary cilia, and the integrin-cytoskeleton system. These sensors activate signaling networks involving Ca^2+^, Rho GTPases, MAPKs, and YAP/TAZ, which regulate adaptive responses or pathological injury (Clearman et al., 2023; Miyano et al., 2022; Steichen et al., 2022; He et al., 2022; Bocanegra et al., 2014; Piccolo et al., 2023). Most studies, however, have emphasized pathological outcomes such as inflammatory infiltration, apoptosis, and renal interstitial fibrosis. Mechanical stress has therefore often been viewed mainly as a cause of renal damage. Its effects on the tubule are in fact bidirectional. Early low-intensity or acute stimulation does not simply cause injury. RTECs can initiate several protective compensatory mechanisms, including cytoskeletal remodeling, regulation of ion transport, and maintenance of proteostasis. These adaptations limit mechanical damage, preserve physiological homeostasis, and support cell survival during stress. Crosstalk between endoplasmic reticulum stress and autophagy may also mediate the transition from acute kidney injury to chronic kidney disease (Habshi et al., 2023). Several molecular pathways participate in tubular adaptation to mechanical stress. Stimuli such as acute ureteral obstruction can regulate heat shock protein phosphorylation, activate WNK4-SPAK/OSR1 signaling, promote WNK body formation, and alter Kelch-like protein 3/Cullin 3-mediated protein degradation. Together, these mechanisms coordinate tubular stress responses and injury repair while maintaining cellular homeostasis (Thomson et al., 2020; Carlsen et al., 2013; Maeoka et al., 2024; Elvers et al., 2022; Maeoka et al., 2022).

Although protective adaptations to mechanical stimulation have begun to emerge, the critical conditions under which individual protective pathways fail remain undefined. The dynamic shift from physiological compensation to persistent, irreversible injury has not been systematically characterized. In tubular models of mechanical stress, the specific triggers, regulatory nodes, and molecular interaction networks that drive this transition are still unclear. Defining these bidirectional responses and the molecular nodes that switch adaptation to injury may clarify the pathogenesis of obstructive nephropathy and other mechanically mediated kidney diseases.

RTECs differ across nephron segments in transport function, embryonic origin, and the mechanical loads they experience. These differences raise a central question: does segmental heterogeneity reflect divergent mechanosensing machinery, or context-dependent deployment of a shared machinery? Current evidence supports a two-tier framework. The identity of the core mechanosensing machinery, including Piezo1, TRPV4, and primary cilia, is highly conserved across nephron segments (Ross et al., 2021; Lapin et al., 2023; Doi et al., 2023; Khalil et al., 2023; Hiratsuka et al., 2022; Gabbin et al., 2023; Nerger et al., 2024; Myram et al., 2021). What varies segmentally is the deployment of this machinery: its abundance, activation thresholds, subcellular localization, downstream coupling, and functional readout are tuned by the local mechanical and metabolic microenvironment (Choudhury et al., 2022; Adapala et al., 2021; Zhao X. et al., 2022; Mamenko et al., 2015). For example, Piezo1 and TRPV4 are expressed along the entire nephron, but flow-dominated sensing prevails in proximal segments, whereas pressure- and volume-dominated sensing prevails in distal segments (Zhao X. et al., 2022; Mamenko et al., 2015). This review therefore treats shared mechanosensing principles as its backbone and addresses segmental heterogeneity explicitly where it determines adaptive strategy or injury predisposition.

The evidence synthesized here integrates human and animal data as well as *in vitro* and *in vivo* findings, and the reader should note the provenance of each line of evidence. Protein expression and localization data derive mainly from immunostaining and immunoblotting of human kidney tissue and rodent nephrons; transcript-level observations are indicated where they have not been confirmed at the protein level. Functional data come from *in vitro* mechanical loading systems, including immortalized human proximal tubular HK-2 cells, primary mouse proximal tubular cells, and rat NRK-52E cells, and from *in vivo* models, most prominently unilateral ureteral obstruction (UUO) in rodents. Because mechanosensitive signaling can show quantitative and even qualitative divergence between accelerated rodent models and chronic human kidney disease, species and experimental context are stated throughout the review wherever they are relevant to interpretation.

The review is organized as follows. Section 2 describes the mechanosensing systems of RTECs, including mechanosensitive ion channels, primary cilia, and the integrin-cytoskeleton axis. Section 3 summarizes the intracellular mechanotransduction pathways (Ca^2+^, Rho GTPases, MAPK cascades, and YAP/TAZ) and their crosstalk. Section 4 examines early adaptive responses and their segmental heterogeneity. Section 5 analyzes the transition from adaptation to pathological injury, including the quantitative and contextual thresholds that govern this transition. Section 6 reviews *in vitro*, *in vivo*, and emerging models and technologies. Section 7 concludes with unresolved questions and future directions, including the translational challenge of phase-dependent targeting.

## 2 Mechanosensing systems in RTECs

RTECs occupy a complex microenvironment shaped by multiple physical forces. Fluid shear stress arises from friction between luminal tubular fluid and the tubular wall. Its magnitude depends on luminal diameter and flow velocity and is particularly prominent in the proximal tubule (Ferrell et al., 2019; Choudhury et al., 2022). Hydrostatic pressure derives from glomerular filtration pressure and the transtubular pressure gradient. It acts directly on the basolateral membrane and fluctuates with resistance along different tubular segments (Choudhury et al., 2022; Adapala et al., 2021; Gilmer et al., 2018). Respiratory motion, cardiac pulsation, and vasomotion are also transmitted to the renal parenchyma, exposing tubules to cyclic stretch. The frequency and amplitude of this stretch follow physiological rhythms (Ferrell et al., 2019; Choudhury et al., 2022). In addition to fluid-derived forces, tubular cells respond to extracellular matrix stiffness. Matrix stiffness varies with interstitial collagen deposition and crosslinking. It increases substantially during fibrosis and can directly alter cell behavior through integrin signaling. These mechanical parameters become abnormal in disease. Ureteral obstruction or congenital urinary tract stenosis sharply raises intraluminal pressure, reduces fluid shear stress, and increases hydrostatic pressure and stretch (Gilmer et al., 2018; Huang et al., 2023). In polycystic kidney disease, expanding cysts compress adjacent tubules and alter the local distribution of tension (Clearman et al., 2023). High-irrigation states, such as those following ureteroscopy or percutaneous nephrolithotomy, produce abnormal pulsatile changes in shear stress and pressure (Hadpech and Thongboonkerd, 2024; Cao et al., 2023). Together with the progressive increase in matrix stiffness, these pathological changes create a proinflammatory and profibrotic mechanical microenvironment. Moreover, these mechanical parameters are dynamically modulated by filtration and reabsorption. Glomerular hyperfiltration in early diabetic nephropathy or after nephron loss increases tubular flow and luminal volume, raising shear stress and wall tension along the proximal tubule (Chagnac et al., 2019; Vallon and Thomson, 2020). Severe interstitial inflammation or edema elevates interstitial hydrostatic pressure and compresses tubular walls, while visceral adiposity and elevated intra-abdominal pressure impose additional extrinsic mechanical loads (Mende and Einhorn, 2022; Abe et al., 2023). These scenarios underscore that mechanical overload of RTECs can arise without anatomical obstruction and extend the clinical relevance of the mechanosensing machinery discussed below.

Mechanical stimuli must first be detected before they can be converted into intracellular biochemical signals and elicit physiological or pathological responses (Adapala et al., 2021; Clearman et al., 2023). RTECs possess a mechanosensory network composed of mechanosensitive ion channels, primary cilia, and integrin-cytoskeleton complexes (Table 1).

**TABLE 1:** Major mechanosensors in RTECs and their functions.

Mechanical stimulus	Sensor	Main segment	Key signals	Adaptive effects	Pathological effects	Associated diseases
Fluid shear stress	Primary cilium; TRPV4; PC1/PC2	Distal tubule; collecting duct	Ca^2+^; cAMP/PKA; AMPK	Maintains polarity; metabolic homeostasis; adaptation to urine flow	Metabolic dysfunction; abnormal proliferation; mild inflammation	Polycystic kidney disease; urine flow-related kidney injury
Elevated transmural hydrostatic pressure	Piezo1; cytoskeleton	Distal tubule; collecting duct	Ca^2+^; YAP/TAZ; NF-κB	Electrolyte secretion; tolerance of pressure fluctuations	Ca^2+^ overload; inflammation; tubular injury	Ureteral obstruction; kidney injury after high-pressure irrigation
Cyclic tissue stretch	Integrins; Piezo1; RhoA/ROCK	All tubular segments (mainly distal)	MAPK; FAK/Src; YAP/TAZ	Cytoskeletal remodeling; tissue elasticity	Cytoskeletal disruption; EMT; apoptosis	Obstructive nephropathy; hydronephrosis
Increased matrix stiffness	Integrin-FAK; YAP/TAZ	Proximal tubule	RhoA/ROCK; TGF-β/Smad	Matrix adaptation; stable cell adhesion	Mechanical memory; ECM accumulation; fixed fibrosis	Chronic kidney disease; renal interstitial fibrosis

Individual sensors show some specificity for different types of mechanical stimulation. They also cooperate extensively to detect, integrate, and amplify mechanical signals (Nauli et al., 2003; Xiao et al., 2023). These sensors are broadly expressed along the nephron, but their activation thresholds, abundance, and downstream effects vary with the local microenvironment and cellular function. This segmental variation provides a structural basis for heterogeneous tubular mechanical responses.

### 2.1 Mechanosensitive ion channels

Mechanosensitive ion channels are the fastest molecular sensors of mechanical stimulation in RTECs. Within milliseconds, they detect changes in membrane tension, fluid shear stress, and osmotic pressure. They rapidly mediate the transmembrane movement of ions such as Ca^2+^, Na^+^, and K^+^, thereby initiating downstream signaling (Mamenko et al., 2015; Song et al., 2022). Channel subtypes are differentially expressed along the tubule and support segment-specific mechanosensing (Figure 1).

**FIGURE 1:**
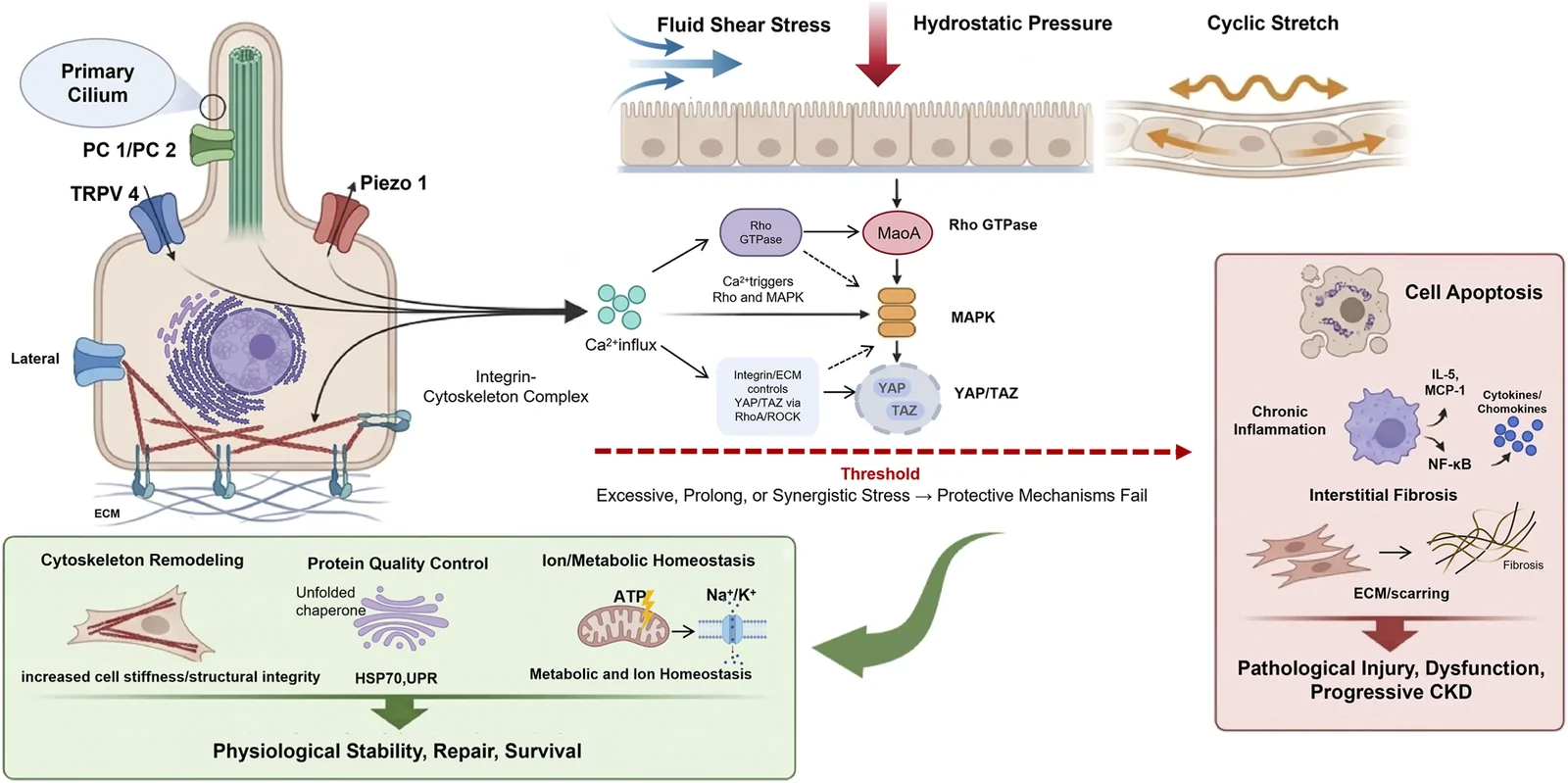
Mechanotransduction and bidirectional cell-fate responses in RTECs under mechanical stress. RTECs perceive three major mechanical cues (luminal fluid shear stress, transmural hydrostatic pressure and cyclic tissue stretch) via three core mechanosensing machineries, which converge onto the Ca^2+^-Rho GTPase-MAPK-YAP/TAZ signaling cascade. Subthreshold physiological mechanical stimuli drive reversible homeostatic adaptation, while persistent over-threshold mechanical stress switches cell fate toward apoptotic death, interstitial inflammation and progressive renal fibrosis.

#### 2.1.1 TRPV4

Transient receptor potential vanilloid 4 (TRPV4) is one of the best-characterized mechanosensitive ion channels in the kidney. It is widely expressed in the proximal tubule, thick ascending limb, distal tubule, and collecting duct (Adapala et al., 2021; Cox et al., 2024; Tomilin et al., 2016). TRPV4 senses fluid shear stress, mechanical stretch, cell swelling, and changes in osmotic pressure. Its activation promotes Ca^2+^ influx and regulates cytoskeletal remodeling, albumin endocytosis, reactive oxygen species production, and inflammatory signaling. These functions contribute to tubular ion homeostasis and mechanical adaptation (Kumar et al., 2022; Wang et al., 2025). In the distal nephron, TRPV4 is more sensitive to osmotic pressure and stretch. It mainly regulates distal tubular K^+^ secretion and osmotic balance (Mamenko et al., 2015). In the proximal tubule, TRPV4 primarily responds to high fluid shear stress and adjusts reabsorptive function. Persistent overactivation of TRPV4 can activate TGF-β, NF-κB, and MAPK signaling. It promotes epithelial-mesenchymal transition (EMT), inflammation, and extracellular matrix deposition, providing an important mechanism by which mechanical stimulation drives renal fibrosis (Ren et al., 2023; Grabias and Konstantopoulos, 2014).

#### 2.1.2 Piezo1

Piezo1 is a large mechanically gated cation channel and a major focus of renal mechanobiology (Zhao X. et al., 2022; Drobnik et al., 2024; Liu et al., 2023). It directly detects changes in plasma membrane tension. Restoration of membrane curvature and altered lipid bilayer tension rapidly open the channel, allowing the initial conversion of a mechanical signal without second messengers (Zhao X. et al., 2022; Drobnik et al., 2024). Piezo1 is expressed along the entire nephron and is not confined to distal segments: in human kidneys, Piezo1 protein is detected in proximal tubular cells as well as in collecting duct principal cells (Zhao X. et al., 2022), and Piezo1 transcript and protein are found in rodent proximal tubules and proximal tubular cell lines (Drobnik et al., 2024). Its abundance is relatively higher in the distal tubule and collecting duct, where it is enriched at the basolateral membrane, than in the proximal tubule (Choudhury et al., 2022; Drobnik et al., 2024). In the distal nephron, Piezo1 detects increased wall tension and tubular dilation. It mediates flow-dependent K^+^ secretion and supports urine concentration and electrolyte regulation (Choudhury et al., 2022). In the collecting duct, Piezo1 is expressed mainly in principal cells (PCs) and only at very low levels in intercalated cells (ICs). This distribution contributes to heterogeneous stretch responses and injury (Zhao X. et al., 2022; Drobnik et al., 2024). Mechanical activation of Piezo1 rapidly increases Ca^2+^ influx and subsequently activates PI3K/Akt, NF-κB, and YAP/TAZ signaling. These pathways regulate inflammation, cell proliferation, and fibrosis (Liu et al., 2023).

In rodent models (unilateral ureteral obstruction and folic acid nephropathy), sustained Piezo1 activation promotes tubular injury and renal interstitial fibrosis, whereas pharmacological inhibition or knockdown of Piezo1 reduces collagen deposition and tissue remodeling. Piezo1 has therefore emerged as a potential therapeutic target in mechanically mediated kidney disease (Drobnik et al., 2024; Liu et al., 2023).

#### 2.1.3 The polycystin complex (PC1/PC2)

Polycystin-1 (PC1) and polycystin-2 (PC2) form a mechanosensitive complex on the primary ciliary membrane and contribute to tubular fluid-flow sensing (Clearman et al., 2023; Nauli et al., 2003; Kim, 2023; Zhang et al., 2023). The complex is enriched in the distal tubule and collecting duct, the segments most prone to cystic lesions (Clearman et al., 2023). When fluid shear stress bends the primary cilium, PC1 undergoes a conformational change and activates PC2-mediated Ca^2+^ influx. The resulting intracellular Ca^2+^ signal regulates cell proliferation, polarity, and nephron development (Nauli et al., 2003; Kim, 2023; Zhang et al., 2023; Peyronnet et al., 2012). Loss of PC1 or PC2 impairs mechanosensing and prevents normal responses to fluid-dynamic signals. The resulting abnormal tubular proliferation and cyst expansion are central molecular features of autosomal dominant polycystic kidney disease (Clearman et al., 2023; Nauli et al., 2003; Kim, 2023).

### 2.2 Primary cilia as mechanical signal-integration centers

The primary cilium of a renal tubular epithelial cell is nonmotile but highly sensitive to mechanical forces (Clearman et al., 2023). Ciliary distribution differs markedly among nephron segments. Proximal tubules have fewer and shorter cilia and rely more heavily on microvilli for mechanosensing, resulting in weaker detection of fluid shear stress. Distal tubules and collecting ducts have long, well-developed cilia that integrate fluid-dynamic signals (Ferrell et al., 2019; Clearman et al., 2023). As major shear-stress sensors, primary cilia convert flow-derived mechanical cues into intracellular biochemical signals. They directly regulate energy homeostasis in RTECs, ensuring the energy supply required for reabsorption. They also integrate mechanical, chemical, metabolic, and growth-factor signals to fine-tune cellular function (Clearman et al., 2023; Nauli et al., 2003; Zhang et al., 2023).

Cilia-mediated Ca^2+^ signaling can activate adenylyl cyclase 6 (AC6), regulate the cAMP-PKA cascade, and alter COX2 expression and inflammatory activity. This signaling axis also modulates canonical developmental and profibrotic pathways, including TGF-β/Smad, BMP, and Hedgehog signaling. Mechanical effects can thus extend from local signaling events to cell-wide transcriptional programs (Kim, 2023; Peyronnet et al., 2012; Sato et al., 2003; Nagashima et al., 2022).

Under pathological conditions, structural or functional abnormalities of primary cilia markedly reduce the ability of cells to sense fluid shear stress. Disrupted mechanical signaling then promotes abnormal proliferation, cystic expansion, and fibrosis. This mechanism is particularly evident in polycystic kidney disease and chronic kidney disease (Clearman et al., 2023; Nauli et al., 2003; Kim, 2023; Zhang et al., 2023; Chen et al., 2020). Beyond mechanosensing, primary cilia also regulate cellular metabolism and energy homeostasis through the mTOR, AMPK, and YAP/TAZ axes. They therefore have a central role in mechanometabolic coupling (Jun et al., 2022; Claude-Taupin et al., 2023; Zuo et al., 2022).

### 2.3 The integrin-cytoskeleton mechanotransduction axis

Integrins are transmembrane receptors that connect the extracellular matrix to the intracellular cytoskeleton. They are key structures through which cells sense matrix stiffness and external stretch (Hamzeh et al., 2015; Xie et al., 2023). In RTECs, their extracellular domains bind matrix proteins such as collagen and fibronectin. Their intracellular domains interact with adaptor proteins, including talin, vinculin, and paxillin, to form focal adhesion complexes. These complexes establish a transmembrane pathway for mechanical force transmission (Hamzeh et al., 2015; Xie et al., 2023).

Mechanical stimulation induces integrin clustering and focal adhesion maturation. These events activate focal adhesion kinase (FAK) and Src family kinases, followed by RhoA/ROCK signaling, actin reorganization, and changes in cell shape (Xie et al., 2023; Zhu et al., 2025; Rogg et al., 2023). This response increases resistance to mechanical stress. Through the LINC complex, it also transmits cytoskeletal forces to the nucleus and regulates chromatin architecture and gene transcription. The resulting mechanical memory preserves a record of previous mechanical stimulation and may contribute to the sustained progression of chronic kidney disease (Moya and Halder, 2019; Drake et al., 2022; Ren et al., 2021).

Integrin signaling also interacts closely with YAP/TAZ. Changes in matrix stiffness regulate YAP nuclear translocation through the integrin-FAK-RhoA axis, thereby influencing cell proliferation, apoptosis, and fibrosis (Mu et al., 2024; Grenier et al., 2024; Beamish et al., 2017; Kapetanaki et al., 2022). Integrins are more abundant in proximal tubular cells, which are consequently more sensitive to matrix stiffness. During fibrosis, these cells are also more prone to cytoskeletal remodeling and EMT. The pathway is less active in distal tubules and collecting ducts, where the predominant response involves regulation of ion homeostasis (Hamzeh et al., 2015).

RTECs therefore detect and transduce external physical stimuli through mechanosensitive ion channels, primary cilia, and integrin-cytoskeleton complexes. These systems are functionally distinct but highly coordinated, together forming an integrated mechanosensory network. Mechanosensitive ion channels mediate rapid electrochemical conversion, whereas primary cilia integrate fluid-dynamic signals. Integrins connect the extracellular matrix to the nucleus and support long-term transmission and retention of mechanical signals. Together, these sensors provide the basis for downstream Ca^2+^ signaling, MAPK cascades, and YAP/TAZ-dependent transcription (Kim, 2023; Mu et al., 2024).

## 3 Intracellular mechanotransduction pathways

Once mechanical stimuli are detected by cell-surface mechanosensors, they do not alter cellular function directly. Instead, several intracellular pathways amplify and integrate these signals before regulating gene expression and cellular state. Ca^2+^ is the earliest second messenger activated. Rho GTPases, MAPKs, and YAP/TAZ form the wider mechanotransduction network, with extensive crosstalk among pathways (Grabias and Konstantopoulos, 2014; Xie et al., 2023). The intensity, duration, and combination of pathway activation determine whether cells undergo adaptive repair or pathological injury.

### 3.1 Ca^2+^ influx initiates intracellular mechanotransduction

Opening of mechanosensitive channels produces an immediate rise in intracellular Ca^2+^ and initiates downstream signaling. In RTECs, this Ca^2+^ transient mainly reflects extracellular influx through TRPV4, Piezo1, and PC2. Fluid shear stress, mechanical stretch, and increased membrane tension can open these channels and rapidly drive extracellular Ca^2+^ into the cell (Cox et al., 2024; Liu et al., 2023). The rise in intracellular Ca^2+^ promotes binding to calmodulin and formation of a Ca^2+^-CaM complex. This complex then activates effectors such as CaMKII, calcineurin, and PKC, converting mechanical stimulation into downstream signaling responses (Miyano et al., 2022; Zuo et al., 2022).

Different patterns of Ca^2+^ signaling have markedly different biological effects, with clear dependence on amplitude and duration. Transient, low-amplitude, rhythmic Ca^2+^ signals induced by physiological shear stress or mild stretch help maintain cell polarity, ion transport, and cytoskeletal stability. They also activate repair-associated genes and represent a typical physiological adaptation. In contrast, sustained, high-intensity Ca^2+^ overload during high pressure, prolonged stretch, or obstruction causes mitochondrial dysfunction and excessive reactive oxygen species (ROS) production. It suppresses repair, promotes apoptosis and inflammation, and initiates pathological injury (Yamamoto et al., 2002; Shu et al., 2022). Ca^2+^ signaling also regulates YAP/TAZ activity and NF-κB-dependent inflammation in a bidirectional manner. It therefore links mechanical adaptation with metabolism, inflammation, and fibrosis (Zuo et al., 2022; Beamish et al., 2017).

### 3.2 Rho GTPase-mediated cytoskeletal remodeling

The rise in Ca^2+^ after mechanical stimulation can activate small Rho-family GTPases, including RhoA, Rac1, and Cdc42. RhoA is widely involved in tubular mechanical responses (Hamzeh et al., 2015; Xie et al., 2023; Zhu et al., 2025). Activated RhoA signals through ROCK to promote actin polymerization and stress-fiber formation. This rapid cytoskeletal remodeling increases cellular resistance to external deformation (Xie et al., 2023). RhoA also promotes focal adhesion formation and stabilizes cell-matrix attachment, allowing mechanical signals to continue propagating intracellularly (Hamzeh et al., 2015).

RhoA-dependent stress fibers connect to the nuclear envelope through the LINC complex and transmit mechanical signals directly to the nucleus. This changes nuclear architecture and chromatin accessibility, regulates mechanoresponsive gene transcription, and establishes mechanical memory. Pathological transcriptional marks may therefore persist after the original stimulus is removed (Moya and Halder, 2019). Moderate RhoA activation drives reversible cytoskeletal remodeling and supports adaptation to mechanical stress. Persistent overactivation instead causes irreversible cytoskeletal disorganization and promotes proliferation, EMT, and renal interstitial fibrosis (Drake et al., 2022).

### 3.3 MAPK cascades

MAPK signaling is a canonical intracellular response to mechanical stimulation. Its three major branches, ERK1/2, JNK, and p38 MAPK, have distinct functions and collectively influence cell fate (Bocanegra et al., 2014; Kurtzeborn et al., 2022). Mechanical stretch rapidly activates membrane-associated phospholipases and promotes ERK1/2 phosphorylation. Short-term, mild stimulation preferentially activates ERK1/2, which regulates proliferation, injury repair, and cytoskeletal homeostasis and thereby supports protective adaptation (Bocanegra et al., 2014). JNK and p38 MAPK are more sensitive to pathological mechanical stimuli. Sustained high pressure or intense stretch causes prolonged phosphorylation of both kinases, which mainly regulate inflammation, oxidative stress, and apoptosis (Bocanegra et al., 2014; Kumar et al., 2022).

*In vitro*, cyclic stretch induces inflammatory mediators such as IL-8 through protein kinase C- and MAPK-dependent signaling and activates NF-κB, thereby amplifying inflammation (Hamzeh et al., 2015; Yamamoto et al., 2002). ERK1/2-dependent repair and JNK/p38-dependent injury remain in dynamic balance. Disruption of this balance is an important marker of the transition from adaptation to injury (Grabias and Konstantopoulos, 2014).

### 3.4 YAP/TAZ regulation of mechanically responsive gene expression

Yes-associated protein (YAP) and transcriptional coactivator with PDZ-binding motif (TAZ) are transcriptional coactivators in the Hippo pathway and act as mechanosensitive transcriptional regulators. In RTECs, their subcellular distribution and transcriptional activity are tightly controlled by cytoskeletal tension and extracellular matrix stiffness. YAP/TAZ therefore link mechanical stimulation to gene expression, mediate mechanical memory, and influence cell fate (Moya and Halder, 2019). Unlike conventional growth factor-dependent transcription factors, they can directly sense changes in the cellular mechanical microenvironment and convert physical cues into transcriptional signals (Moya and Halder, 2019).

At rest, the Hippo kinase cascade remains active. LATS1/2 phosphorylates YAP/TAZ, retains them in the cytoplasm, and promotes their ubiquitin-mediated degradation, thereby maintaining low basal transcriptional activity (Moya and Halder, 2019). Moderate mechanical stimulation increases cytoskeletal tension and suppresses Hippo signaling. YAP/TAZ are then dephosphorylated and translocate to the nucleus, where they bind TEAD-family transcription factors. This complex induces repair-associated genes such as CTGF and CYR61, promotes proliferation, preserves epithelial integrity, and supports protective repair after acute injury (Ren et al., 2021).

Persistent high-intensity stimulation and increased matrix stiffness cause prolonged nuclear activation of YAP/TAZ and disrupt physiological homeostasis. Profibrotic genes such as COL1A1 and FN1 are strongly induced, promoting extracellular matrix deposition and fibroblast activation. These changes drive irreversible renal interstitial fibrosis (Chen et al., 2020; Drake et al., 2022). TGF-β also promotes YAP/TAZ nuclear translocation, while activated YAP/TAZ further enhance Smad transcriptional activity. This feed-forward loop sustains profibrotic signaling and contributes to mechanical memory and chronic injury progression (Ren et al., 2023; Sato et al., 2003). In addition, YAP/TAZ regulate autophagy and energy homeostasis through the AMPK-SIRT1 axis. They therefore participate in early metabolic adaptation while exerting both protective and pathological effects (Claude-Taupin et al., 2023).

The dual protective and pathological functions of YAP/TAZ raise a central therapeutic question: if YAP/TAZ inhibition is considered as an anti-fibrotic strategy, how can its physiological repair functions be preserved? Available data indicate that the net effect of YAP/TAZ modulation is phase-dependent. Transient, moderate YAP/TAZ nuclear activity supports protective repair and epithelial integrity after acute injury (Moya and Halder, 2019; Ren et al., 2021), whereas persistent nuclear retention during the fibrotic phase drives matrix deposition (Chen et al., 2020; Drake et al., 2022). Non-selective or long-term inhibition therefore risks blunting the early adaptive response, whereas late, transient, or segment-targeted inhibition during established fibrosis may retain antifibrotic benefit with less harm to repair. Timing strategies could include short-course regimens initiated after fibrosis is established, combination with repair-promoting agents, and biomarker-guided monitoring of YAP/TAZ activity to position the intervention along the adaptation-injury continuum (Table 2). These phase-dependent considerations apply equally to other mechanically activated targets whose inhibition is protective in the pathological phase.

**TABLE 2:** Potential therapeutic targets for mechanically mediated kidney injury and barriers to clinical translation.

Class	Target	Pathway role	Intervention	Stage	Indications	Limitations
Channel	Piezo1	Mechanical sensing; initiates Ca^2+^ influx	GsMTx4; selective small-molecule inhibitors	Cell and animal studies	Obstructive nephropathy; interstitial fibrosis; polycystic kidney disease	May disturb electrolyte balance; poor targeting and stability; no oral small-molecule candidate
Channel	TRPV4	Ca^2+^ signaling; inflammation; upstream EMT switch	HC-067047; selective TRPV4 antagonists	Cell and animal studies	Obstructive nephropathy; hypertensive kidney injury; cyst expansion in polycystic kidney disease	Long-term inhibition may disrupt tubular homeostasis; systemic expression raises blood-pressure and edema risks; no kidney-selective inhibitor
Cytoskeletal	RhoA/ROCK	Cytoskeletal remodeling; upstream YAP control; stress-fiber formation	Fasudil, hydroxyfasudil, and other ROCK inhibitors	Approved for cerebrovascular indications	Obstructive renal fibrosis; chronic mechanical stress-related kidney disease	Low renal exposure requires high doses; circulatory adverse effects; limited antifibrotic efficacy with monotherapy
Gene regulation	YAP/TAZ	Core downstream coactivators; profibrotic transcription	Verteporfin; TEAD-binding inhibitors	Animal-model validation	Mechanically induced interstitial fibrosis; obstructive nephropathy	Phase-dependent: Inhibition during the early adaptive phase may impair repair and epithelial regeneration, whereas transient inhibition during established fibrosis may retain antifibrotic benefit; limited kidney specificity; systemic effects; cannot reverse established matrix stiffening or epigenetic memory
Proteostasis	HSP70	ERS and misfolded-protein repair; early stress protection	HSP70 inducers; recombinant HSP70	*In vitro* cell studies	Acute mechanical tubular injury; early obstructive stress	Narrow intervention window; poor delivery and rapid degradation; proliferation risk may conflict with polycystic kidney disease therapy

### 3.5 Pathway crosstalk and dynamic balance

Intracellular mechanotransduction in RTECs is not a simple combination of linear pathways. It forms a highly interconnected network under dynamic control, and the balance among signals determines whether cells adapt or sustain injury (Grabias and Konstantopoulos, 2014). Mechanosensitive channels first trigger a rapid Ca^2+^ transient. This initiating signal activates downstream effectors such as CaMK and PKC and rapidly engages RhoA-dependent cytoskeletal remodeling, providing early structural adaptation (Livingston et al., 2024). The remodeled cytoskeleton then regulates YAP/TAZ nuclear localization and converts a transient mechanical cue into sustained transcriptional control (Beamish et al., 2017).

Under short-term, low-intensity physiological stimulation, protective signals predominate. Moderate ERK1/2 activation, transient YAP/TAZ activity, and stable Ca^2+^ oscillations support cytoskeletal repair, ion homeostasis, and metabolic adjustment. These responses permit reversible adaptation. Under sustained, high-intensity pathological stimulation, Ca^2+^ overload and excessive RhoA activation occur together with persistent JNK/p38 and NF-κB signaling. Pathological YAP/TAZ nuclear retention, ROS accumulation, and uncontrolled endoplasmic reticulum stress further reinforce the network. The resulting signaling cascade drives cells from physiological adaptation toward apoptosis, inflammation, and fibrosis (Grabias and Konstantopoulos, 2014). Inflammatory and oxidative signals can also increase mechanosensitive channel activity, further amplifying mechanical stress. This vicious cycle provides a molecular basis for progressive chronic kidney injury. Thus, both the duration and magnitude of pathway activation determine adaptation or injury. No single pathway independently dictates the final cell fate.

## 4 Early adaptive responses to mechanical forces and segmental heterogeneity

Mechanical adaptation is a reversible, protective process through which cells restore homeostasis during short-term or moderate mechanical stimulation. It involves structural remodeling, maintenance of proteostasis, regulation of ion transport, and adjustment of energy metabolism. Together, these responses buffer mechanical damage and increase stress tolerance. When stimulation intensifies, persists too long, or acts within a deteriorating microenvironment, the protective machinery becomes depleted and imbalanced. Cells then shift from adaptation to maladaptation and initiate pathological injury (Hadpech and Thongboonkerd, 2024; Grabias and Konstantopoulos, 2014). Because nephron segments differ in structure and function, their adaptive responses are markedly heterogeneous.

### 4.1 Cytoskeletal remodeling and mechanical protection

Cytoskeletal remodeling is among the earliest protective responses to mechanical stimulation in RTECs. Rapid activation of RhoA/ROCK promotes actin polymerization and stress-fiber formation. The resulting increase in cellular stiffness reduces direct damage to the plasma membrane and organelles from external stretch and pressure (Xie et al., 2023). Mechanical stimulation also promotes focal adhesion maturation and stabilizes integrin-mediated attachment to the extracellular matrix. Forces are then distributed more evenly, preventing structural injury caused by local stress concentration (Hamzeh et al., 2015). Coordinated reorganization of microtubules, intermediate filaments, and actin maintains cell polarity, intercellular junctions, and the tubular barrier (Xie et al., 2023).

This response differs among nephron segments. Proximal tubular cells rely mainly on dynamic actin remodeling to accommodate frequent fluctuations in fluid shear stress and preserve reabsorption. Distal tubular and collecting duct cells primarily reinforce focal adhesions and increase overall stiffness to tolerate sustained hydrostatic pressure and tubular dilation (Ferrell et al., 2019; Hamzeh et al., 2015). Moderate cytoskeletal remodeling is a reversible protective response. Prolonged remodeling, however, retains abnormal mechanical signals and establishes mechanical memory, providing a structural basis for subsequent fibrosis (Moya and Halder, 2019).

### 4.2 Endoplasmic reticulum stress and protein quality control

Changes in mechanical tension can disrupt normal protein folding through physical connections between the cytoskeleton and endoplasmic reticulum, thereby inducing endoplasmic reticulum stress (ERS) (Nakatsuka et al., 2021). To maintain proteostasis, cells rapidly initiate the unfolded protein response (UPR). Its three principal branches, PERK, IRE1, and ATF6, suppress aberrant protein translation, increase molecular chaperone expression, and promote degradation of misfolded proteins. These actions reduce the endoplasmic reticulum burden and protect the cell (Shu et al., 2022; Wu et al., 2023).

During moderate mechanical stimulation, the UPR remains tightly controlled. It corrects protein-folding defects and preserves cellular homeostasis. When stimulation persists beyond the tolerance threshold, chronic ERS cannot be resolved. The UPR then shifts from protection to a proapoptotic program, increases CHOP expression, and activates mitochondrial apoptosis, resulting in irreversible injury (Shu et al., 2022; Wang Z. et al., 2022). Endoplasmic reticulum stress links mechanical stress, oxidative stress, and inflammation. It acts as a critical switch in the transition from acute adaptation to chronic injury (Shu et al., 2022; Chen et al., 2022; Hasegawa and Inagi, 2020).

### 4.3 HSP70 and proteostasis

Heat shock protein 70 (HSP70) is among the earliest protective stress proteins induced by mechanical stimulation and is a component of the protein quality-control system (Nakatsuka et al., 2021; Costantino et al., 2021). When mechanical stress disrupts protein conformation, HSP70 rapidly binds exposed hydrophobic domains in abnormal proteins. It promotes correct folding and repairs damaged proteins. HSP70 also cooperates with the ubiquitin-proteasome system and autophagy to remove irreversibly damaged proteins. These actions suppress protein aggregation and proteotoxicity and help preserve cellular homeostasis (Wang Z. et al., 2022; Kang et al., 2021).

The protection provided by endogenous HSP70 is limited by both time and dose. It can counter only mild, short-term mechanical stress. During sustained high-intensity stimulation, HSP70 expression cannot offset apoptotic and injury signals, and the protective mechanism fails (Nakatsuka et al., 2021; Costantino et al., 2021). Exogenous induction of HSP70 expression can markedly increase cellular tolerance to mechanical stress. HSP70 may therefore provide an early intervention target for mechanically mediated kidney injury.

### 4.4 Maintenance of ion homeostasis and metabolic adaptation

Mechanical stimulation directly alters membrane tension and cell volume and can disrupt the transport of Na^+^, K^+^, Cl^−^, and Ca^2+^. RTECs rapidly compensate by adjusting ion-transport systems. This preserves osmotic and ionic homeostasis and reduces the risk of mechanical injury (Choudhury et al., 2022; Peng and Warnock, 2007). The adaptation is highly segment specific and matches the physiological function of each segment.

WNK4 is predominantly expressed in the distal nephron, where it is localized to the distal convoluted tubule, connecting tubule, and collecting duct, with additional expression in the thick ascending limb (Thomson et al., 2020; Peng and Warnock, 2007; Lin et al., 2022). It acts as a sensor of mechanical and osmotic changes and detects cell volume, intracellular Cl^−^ concentration, and membrane tension. Through the WNK4-SPAK/OSR1 pathway, it regulates ion transporters such as NCC, ENaC, and NKCC2. These adjustments coordinate electrolyte reabsorption and secretion during mechanical stimulation and maintain Na^+^, K^+^, and osmotic balance (Thomson et al., 2020; Maeoka et al., 2024; Peng and Warnock, 2007; Lin et al., 2022; Brown et al., 2021).

Metabolic adaptation also differs among segments. The proximal tubule has high metabolic demand and abundant mitochondrial activity. Fluid shear stress promotes LRRK2-dependent redistribution of mitochondrial polarity and increases ATP production efficiency, supporting the high reabsorptive workload. It also activates autophagy through the AMPK-SIRT1-YAP axis, removing damaged organelles and preserving metabolic homeostasis (Tabibzadeh et al., 2023; Claude-Taupin et al., 2023). Distal tubular and collecting duct cells have lower metabolic activity. They adapt mainly by remodeling ion homeostasis, with a smaller contribution from metabolic regulation.

Moderate Ca^2+^ transients activate key enzymes in the mitochondrial tricarboxylic acid cycle and increase ATP synthesis. This energy supports cytoskeletal remodeling, protein repair, and ion transport. Sustained Ca^2+^ overload instead lowers mitochondrial membrane potential, causes a burst of ROS, and opens the mitochondrial permeability transition pore (mPTP). The resulting metabolic collapse and apoptosis provide a major metabolic basis for the transition from adaptation to injury (Grabias and Konstantopoulos, 2014; Sunami et al., 2004).

Cytoskeletal remodeling, protein quality control, ion homeostasis, and metabolic reprogramming together form an early protective network during mechanical stress. This network is reversible and compensatory but has a defined tolerance threshold. Once mechanical stimulation exceeds the critical load, adaptive mechanisms become progressively imbalanced and depleted. Cells then enter maladaptation and initiate inflammation, apoptosis, and fibrosis (Figure 2).

**FIGURE 2:**
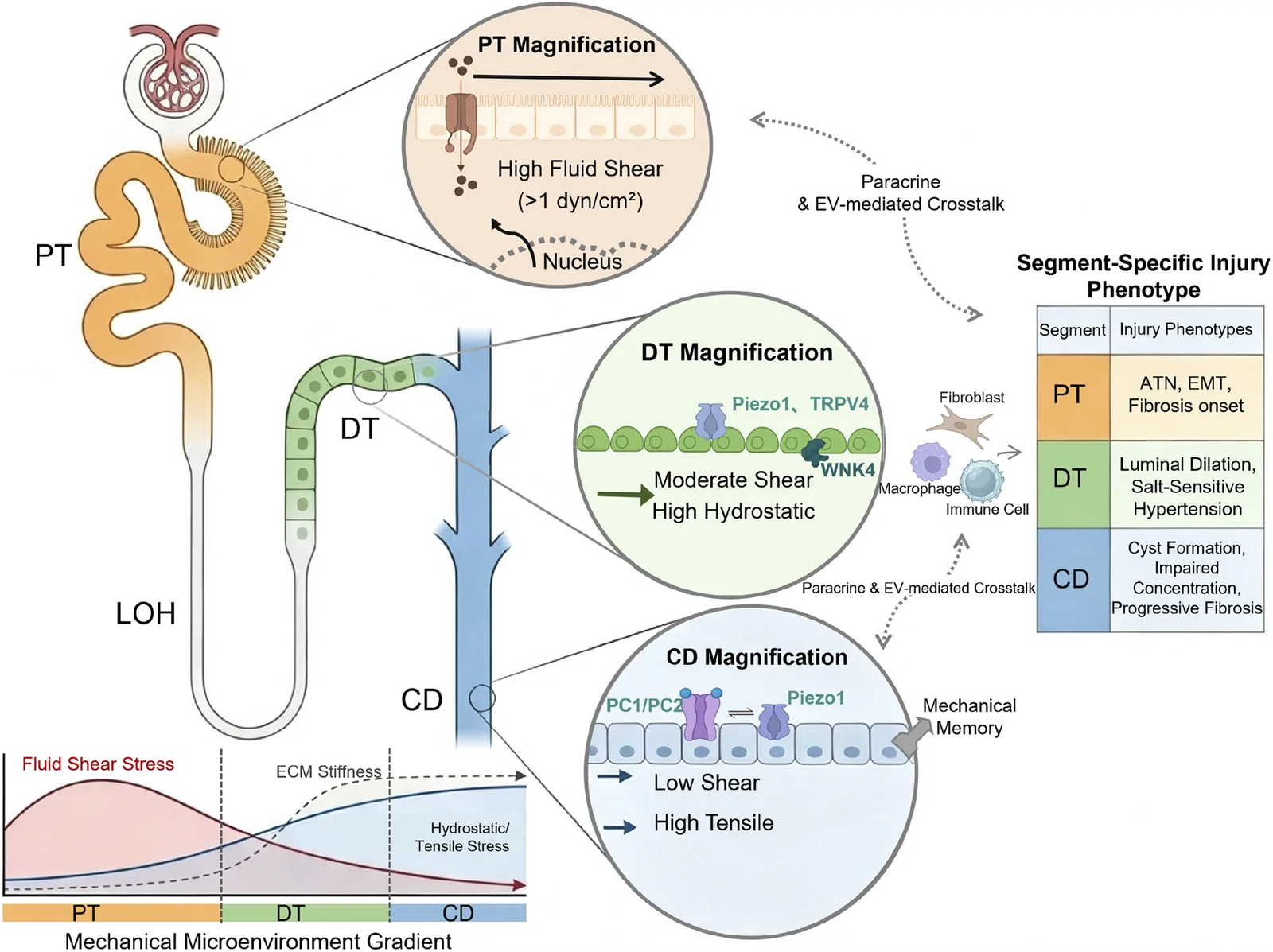
Segment-Specific Mechanosensing, Adaptive Strategies, and Injury Predisposition Along the Nephron. In PT, high fluid shear stress drives metabolic reprogramming and actin dynamics, whereas DT prioritizes WNK4-mediated ion homeostasis under high hydrostatic pressure, and CD relies on primary cilium–polycystin complex (PC1/PC2) and Piezo1 to sense luminal distension. A mechanical microenvironment gradient at the bottom illustrates the relative changes in fluid shear stress, hydrostatic/tensile stress, and ECM stiffness along the nephron axis. Dashed arrows indicate paracrine signaling toward fibroblasts and macrophages, linking segmental tubular responses to the broader inflammatory and fibrotic microenvironment. Abbreviations: PT, proximal tubule; LOH, loop of Henle; DT, distal tubule; CD, collecting duct; ATN, acute tubular necrosis; EMT, epithelial–mesenchymal transition; PC1/PC2, polycystin-1/polycystin-2; ECM, extracellular matrix.

## 5 Transition from mechanical adaptation to pathological injury

Adaptive responses in RTECs can buffer mechanical damage and preserve tissue homeostasis over the short term. This protection is strictly dependent on stimulus intensity, duration, and the surrounding microenvironment. When stimulation remains above the tolerance threshold, or occurs alongside hypoxia, inflammation, or metabolic dysfunction, protective adaptation progressively fails. Cells enter maladaptation, and mechanical signaling is continuously amplified through close coupling with inflammation, oxidative stress, and metabolic disturbance. The eventual outcomes include apoptosis, chronic inflammatory infiltration, and renal interstitial fibrosis. These processes drive the progression of mechanically mediated chronic kidney diseases, including obstructive nephropathy and polycystic kidney disease (Grabias and Konstantopoulos, 2014).

### 5.1 Mechanical stimulation-induced apoptosis

Transient physiological stimulation activates protective repair pathways without marked apoptosis (Hegarty et al., 2002). In contrast, in cultured RTECs, high-intensity stretch lasting more than 6 h or hydrostatic pressure above the tolerance limit can initiate apoptosis (Bocanegra et al., 2014; Grabias and Konstantopoulos, 2014; Li et al., 2009). Prolonged mechanical stress causes sustained Ca^2+^ overload and mitochondrial dysfunction, leading to cytochrome c release. Sequential activation of caspase-9 and caspase-3 then initiates the intrinsic apoptotic program (Bocanegra et al., 2014; Wang Z. et al., 2022; Miyaj et al., 2000). Chronic ERS also increases CHOP expression and promotes transcription of proapoptotic genes through the PERK-eIF2α-ATF4 pathway, further amplifying apoptosis (Shu et al., 2022).

The compensatory capacity of endogenous protective proteins such as HSP70 declines with continued stimulation. These proteins can no longer oppose sustained apoptotic signals, and injury becomes irreversible. Unilateral ureteral obstruction (UUO) models show that adaptive responses predominate during the first 1–3 days of obstruction. Apoptotic signaling increases markedly after day 3 and precedes fibrosis, making it an early event in progressive renal functional decline (Niu et al., 2025).

### 5.2 Formation of a chronic inflammatory microenvironment

Mechanical stress can act as an independent sterile danger signal. It directly activates innate inflammatory pathways without pathogens and promotes a chronic inflammatory microenvironment (Liu et al., 2022; Du et al., 2023). Pathological stimulation activates Piezo1 and TRPV4, causing sustained Ca^2+^ influx and NF-κB activation. Expression of IL-6, IL-8, MCP-1, and TNF-α increases, recruiting macrophages and lymphocytes (Liu et al., 2022; Hamzeh et al., 2015). Persistent activation of the integrin-FAK-Src axis also engages STAT3 and TGF-β signaling. Continued cytokine release then converts an acute stress response into chronic inflammation (Qi et al., 2021).

Mechanical stress also generates large amounts of ROS. Acting as second messengers, ROS further activate NF-κB and MAPK signaling and aggravate tubular injury (Shu et al., 2022). Unlike conventional inflammatory injury, mechanically mediated inflammation begins with a physical signal. Inflammation and mechanical stress then reinforce each other, creating a self-sustaining pathological state.

### 5.3 Mechanical stimulation promotes renal interstitial fibrosis

When mechanical stimulation persists, inflammatory and stress signals eventually drive the kidney into irreversible fibrosis. This process underlies the progression of mechanically mediated kidney disease to end-stage kidney disease (Sato et al., 2003). Persistent mechanical stress increases TGF-β1 expression and activates Smad2/3 signaling, inducing extensive deposition of extracellular matrix proteins such as collagen I, collagen III, and fibronectin (Li et al., 2023; Liu E. et al., 2021; Liu X. et al., 2021). At the same time, sustained YAP/TAZ nuclear localization promotes complex formation with Smad and amplifies transcription of profibrotic genes such as CTGF, accelerating fibrosis (Chen et al., 2020; Drake et al., 2022).

Mechanical memory is an important mechanism in this process. Prolonged mechanical stress regulates chromatin remodeling and epigenetic modifications through the LINC complex, keeping profibrotic genes accessible and active. Progressive matrix stiffening also feeds back to activate integrin-YAP signaling (Moya and Halder, 2019). Even after the initial stimulus is removed, persistent epigenetic marks and a stiffened microenvironment continue to drive fibrosis. This persistence helps explain the irreversibility of chronic kidney disease. Piezo1 and TRPV4 are upstream mechanical targets. Their selective inhibition can interrupt the fibrotic cascade and reduce collagen deposition (Drobnik et al., 2024; Liu et al., 2023).

### 5.4 Combined effects of stimulus intensity, duration, and microenvironment

Existing *in vitro* loading experiments and quantitative animal data begin to delineate, rather than fully define, the critical transition of tubular mechanical responses, and the quantitative values available remain model- and species-specific. The first feature is an intensity threshold. Physiological or low fluid shear stress, including 0.3 dyn/cm^2^, and mild cyclic stretch fall within a safe adaptive range and activate protective responses (Grabias and Konstantopoulos, 2014). For stretch, pathophysiological loading of human proximal tubular cells at approximately 20% elongation for hours to days induces Piezo1-dependent profibrotic responses, whereas shorter or milder regimens are tolerated (Zhao X. et al., 2022; Bocanegra et al., 2014). For pressure, *in vitro* application of 30–100 cmH_2_O (approximately 22–74 mmHg) for 24 h induces myofibroblast transdifferentiation of rat tubular epithelial cells (Li et al., 2009), and in a porcine model, renal pelvic pressure above approximately 20 mmHg causes progressive nephron injury (Wang et al., 2013). The second feature is a duration threshold. Short exposures mainly produce reversible adaptation. With persistent stimulation, protective pathways become imbalanced, apoptotic and inflammatory signaling remains active, and mechanical memory develops. Injury then becomes progressively irreversible (Grabias and Konstantopoulos, 2014). The third feature is a microenvironmental threshold. Hypoxia, high glucose, accumulated inflammatory mediators, and oxidative stress substantially lower cellular tolerance. Mechanical stimuli that would otherwise be physiological can then become pathological, accelerating the transition from adaptation to injury (Hadpech and Thongboonkerd, 2024; Grabias and Konstantopoulos, 2014). Importantly, these thresholds are not universal constants. They differ across nephron segments, because each segment experiences a distinct combination of shear stress, hydrostatic pressure, and stretch (Choudhury et al., 2022; Chagnac et al., 2019), and they are modulated by species, experimental model, and the transcriptional versus protein readout used. Systematic, standardized quantification of these thresholds across segments and models therefore remains an important gap. Nevertheless, existing data show that these thresholds separate physiological adaptation from irreversible injury and determine whether mechanosensitive channels and downstream pathways exert protective or pathogenic effects.

Mechanically mediated kidney injury is therefore a continuous pathological process shaped by several interacting factors. Low-intensity, short-term stimulation in a normal microenvironment supports homeostatic adaptation. High-intensity, prolonged stimulation in an adverse microenvironment causes complete failure of adaptive mechanisms. Apoptosis, chronic inflammation, and irreversible fibrosis then develop progressively and ultimately impair renal function. The combined effects of intensity, duration, and microenvironment mark the boundary between physiological compensation and irreversible injury. They also determine the protective or pathogenic roles of the associated pathways.

### 5.5 Multicellular interactions amplify injury

Earlier studies largely focused on cell-autonomous responses in renal tubular epithelium. *In vivo*, mechanically mediated kidney injury is a coordinated multicellular process involving RTECs, renal interstitial fibroblasts, and macrophages. Their interactions sustain pathological amplification and make fibrosis irreversible (Zhang et al., 2022; Balzer et al., 2022). After sensing abnormal mechanical stimulation, RTECs secrete chemotactic and profibrotic mediators such as MCP-1, IL-6, and TGF-β. These signals recruit circulating macrophages into the renal interstitium. Within the stiffened mechanical microenvironment, infiltrating macrophages undergo polarization and release inflammatory and profibrotic cytokines. They further damage the tubular epithelium and activate quiescent interstitial fibroblasts (Zhang et al., 2022). Activated fibroblasts are highly sensitive to matrix stiffness. Persistent YAP/TAZ activation drives abundant collagen and fibronectin synthesis, causing interstitial stiffening and a further increase in matrix rigidity (Chen et al., 2020). This increased rigidity feeds back to raise the mechanical load on both RTECs and interstitial fibroblasts. The resulting disturbance of renal microenvironmental homeostasis drives continued disease progression.

## 6 Research models and tools

Studies of mechanically mediated kidney injury depend on controllable experimental models and analytical technologies. Combining *in vitro* and *in vivo* systems with emerging methods allows the biological effects of mechanical forces to be examined with greater precision and supports mechanistic investigation.

### 6.1 *In vitro* mechanical loading models

*In vitro* models allow precise control of mechanical parameters and reduce interference from the complex *in vivo* environment. They can therefore isolate the effects of mechanical forces on RTECs. Three model types are widely used. Cell-stretch systems use negative pressure to deform a flexible culture substrate and simulate cyclic or static stretch (Specioso et al., 2022). Fluid shear devices control flow velocity and direction to reproduce the luminal shear environment of renal tubules (Wang D. et al., 2022). Pressure-controlled culture systems adjust hydrostatic pressure within the chamber and recreate a pathological high-pressure renal microenvironment (Li et al., 2009). These models can be used alone or in combination according to the experimental question. They permit precise quantification of stimulus intensity and duration and provide experimental support for studies of critical thresholds.

### 6.2 *In vivo* models

The UUO model is the most established and widely used *in vivo* model of mechanically mediated kidney injury (Niu et al., 2025). Surgical ligation of the ureter rapidly raises intratubular pressure and creates persistent abnormal stretch. The model closely reproduces the pathological course of obstructive nephropathy, including mechanically induced tubular apoptosis, inflammatory activation, and fibrosis. It is therefore indispensable for investigating the pathogenesis of mechanically mediated kidney disease (Niu et al., 2025; Liu E. et al., 2021). Injury severity can be resolved across different obstruction periods, enabling *in vivo* validation of the transition from adaptation to injury.

### 6.3 Emerging technologies and applications

Kidney-on-a-chip platforms use microfluidics to recreate the three-dimensional architecture of renal tubules. They allow precise control of fluid shear stress, hydrostatic pressure, and other mechanical parameters. Segment-specific mechanical microenvironments can therefore be modeled while cellular responses are monitored dynamically in real time. These platforms are promising for studies of mechanical thresholds and segmental heterogeneity (SGaedck et al., 2022; Klocke et al., 2022). Dynamic Ca^2+^ imaging and fluorescence resonance energy transfer (FRET)-based tension sensors can track mechanotransduction in living cells in real time. These methods enable dynamic visualization and quantitative analysis and substantially improve the precision and depth of mechanobiological studies (Grashoff et al., 2010). Single-cell sequencing can resolve differences in mechanical responses among tubular subtypes, interstitial cells, and immune cells. It can also clarify cellular heterogeneity and multicellular interactions (Zhang et al., 2022; Balzer et al., 2022; Schreibing and Kramann, 2022; Wilson et al., 2022; Hasegawa, 2022; Abedini et al., 2024).

## 7 Conclusions and perspectives

RTECs display a complete temporal sequence of responses to mechanical stimulation. They first sense and transduce mechanical signals and then mount a physiological adaptation. The eventual outcome, repair and regeneration or pathological injury, depends on stimulus intensity, duration, and microenvironment. Mechanosensitive ion channels, primary cilia, and the integrin-cytoskeleton axis detect mechanical cues. Ca^2+^ signaling, the Rho-cytoskeleton pathway, and MAPK cascades transmit them intracellularly. Cytoskeletal remodeling, protein quality control, ion homeostasis, and metabolic reprogramming then support physiological adaptation. When persistent high-intensity stimulation exceeds the cellular tolerance threshold, an adverse microenvironment and multicellular interactions further disrupt adaptation. Apoptosis, chronic inflammatory infiltration, and irreversible renal fibrosis ensue, causing permanent loss of renal function.

Mechanical responses vary substantially among tubular segments. Proximal tubules respond strongly to fluid shear stress and adapt mainly through metabolic reprogramming and dynamic cytoskeletal control. Distal tubules emphasize osmotic and ion homeostasis and depend on WNK4 and Piezo1 to accommodate mechanical changes. Collecting ducts use the primary cilium-polycystin complex as a major sensory structure, and abnormal mechanical stimulation readily promotes cystic lesions and fibrosis. Multicellular interactions among fibroblasts, macrophages, and RTECs amplify pathological injury and make fibrosis irreversible. Mechanical memory and epigenetic remodeling provide a molecular basis for the continued progression of chronic kidney disease.

Several important questions remain unresolved. A standardized quantitative system for defining the threshold between cellular adaptation and injury has not been established, and quantitative threshold values remain scattered across models, species, and readouts. The distinct pathways activated by stretch, shear stress, and hydrostatic pressure, as well as their interactions, require further clarification. Transcriptomic and epigenetic heterogeneity among tubular cell subtypes is still poorly understood. Crosstalk between mechanical signaling, inflammation, metabolism, and epigenetic regulation also warrants further study. Finally, the dynamic regulation of multicellular mechanical interaction networks remains unclear. Standardized reporting of loading parameters, including species, cell type, stimulus magnitude and duration, and mRNA versus protein readout, will be essential to make threshold values comparable across studies (Li et al., 2009).

Substantial barriers also limit clinical translation of current mechanical targets. Small-molecule inhibitors of Piezo1 and TRPV4 can produce systemic off-target effects and significant cardiovascular and neurological adverse effects, while kidney-specific delivery methods are lacking. ROCK and YAP inhibitors have shown efficacy in animal models but remain limited by weak potency, high toxicity, and poor clinical suitability. HSP70- and ERS-related targets have been examined only in cell-based experiments, and their *in vivo* stability and safety remain unverified. Importantly, the timing of intervention relative to the adaptation-injury continuum must be considered: for targets such as YAP/TAZ, transient or phase-specific inhibition during established fibrosis may preserve the protective repair functions that operate in the early adaptive phase (Chen et al., 2020; Moya and Halder, 2019). Future work should improve target specificity and delivery systems and develop combination strategies. These efforts may help bridge the gap between basic research and clinical application (Table 2).

Several actionable research directions follow from the gaps identified above. First, kidney-on-a-chip platforms with segment-specific modules should be used to map intensity and duration thresholds for shear stress, pressure, and stretch in each nephron segment under standardized conditions (SGaedck et al., 2022; Klocke et al., 2022). Second, real-time mechanical sensing approaches, including FRET-based molecular tension sensors and intravital imaging, should be applied to track when and where mechanotransduction switches from adaptive to pathological states *in vivo* (Grashoff et al., 2010). Third, single-cell and spatial multi-omics should resolve how tubular subtypes differ in mechanosensor abundance and downstream coupling, linking transcriptomic heterogeneity to functional segmental responses (Zhang et al., 2022; Balzer et al., 2022; Hasegawa, 2022; Abedini et al., 2024). Fourth, phase-specific intervention studies, for example, transient inhibition of YAP/TAZ or Piezo1 during the fibrotic phase, are needed to test whether timing preserves protective repair while limiting fibrosis (Zhao X. et al., 2022; Chen et al., 2020). Fifth, integration of these multi-scale datasets with computational models could support predictive frameworks that estimate individual thresholds from mechanical, metabolic, and inflammatory parameters. These approaches may identify highly specific and safe targets for mechanical intervention and support new strategies that restore normal mechanobiology. Such advances could provide a theoretical basis for precision diagnosis and treatment of obstructive nephropathy, polycystic kidney disease, and chronic renal fibrosis driven by abnormal mechanical forces.
